# Supplementary material for: Neuroprotective Strategies and Cell-Based Biomarkers for Manganese-Induced Toxicity in Human Neuroblastoma (SH-SY5Y) Cells
Source: Biomolecules. 2024 May 31;14(6):647. doi: 10.3390/biom14060647 (PMC11201412; doi:10.3390/biom14060647)
Supplement: Supplementary file 1 [file biomolecules-14-00647-s001.zip › biomolecules-2833118-supplementary.pdf]

APP vs β-actin.

A

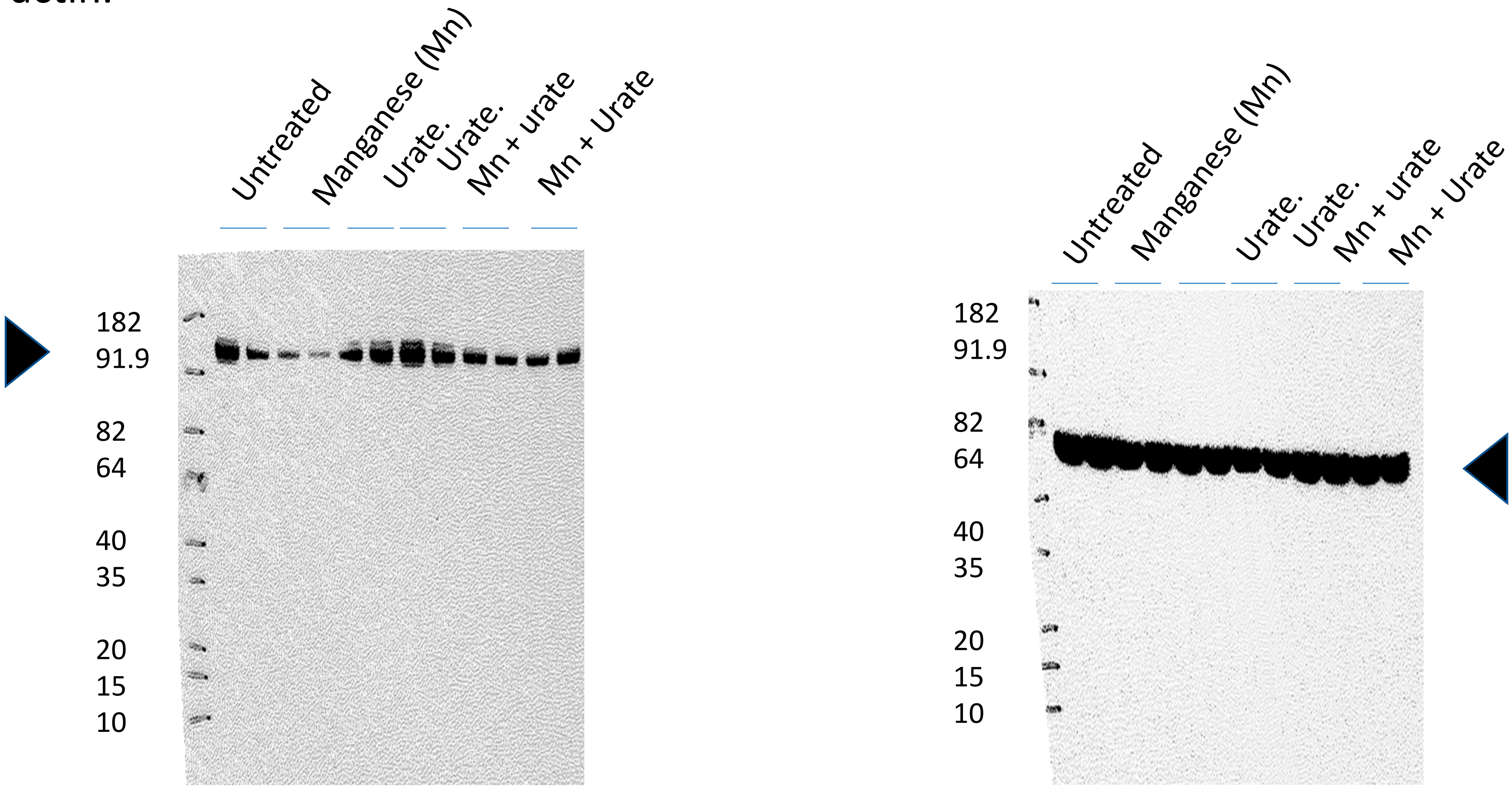

Supplemental Figure 1: Full-length gels for APP (22C11) for figure 3

H-Ferritin vs β-actin.

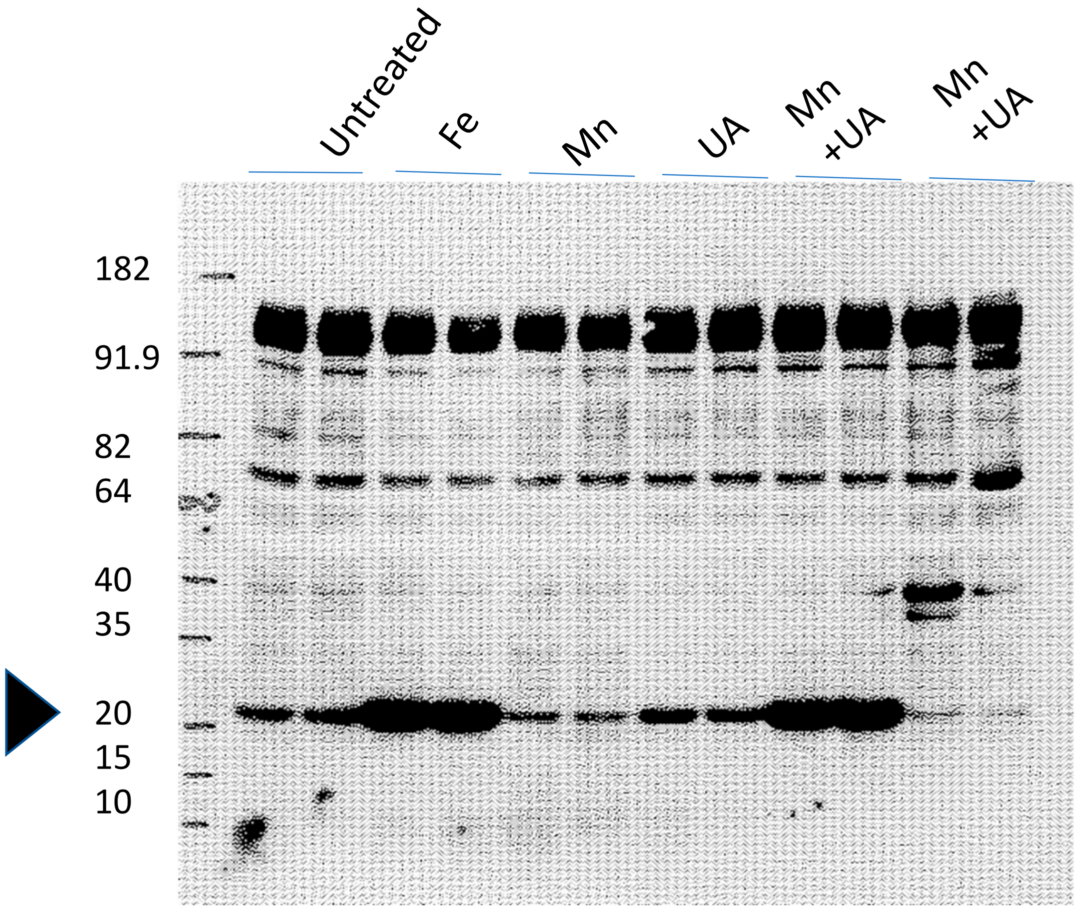

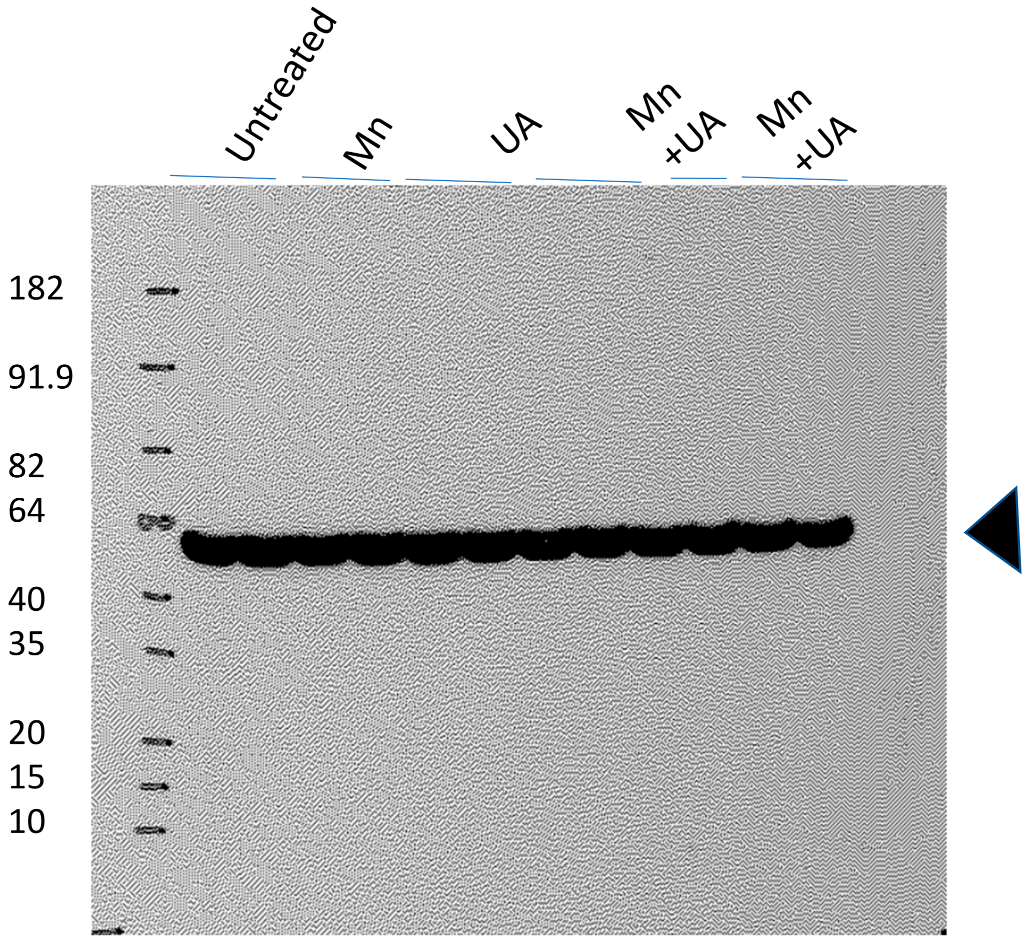

Supplemental Figure 2: Full length gels for H-ferritin for figure 3

```
Human APP mRNA (46)    GGGGCCCCGGGAGACGGCGGCGGTGGCGGCG---CGGGCAGAGCAAGGACGCGGCGGATC   (-43)  AUG
Mouse APP mRNA (33)    GGGGCCACCGGAGACGGCGGCGGCGGCG------CGGACACAGCCAGGGCGCGGCGGATC   (-45)  AUG
Rhesus APP     (137)   GGGTCCCCCGGAGACGGCGGCGGTGGCG------CGGGCAGAGCAAGGACGCGGCGGATC   (-43)  AUG
Bovine APP     (33)    GGGGCCACCGGAGACGGCGGCGGCGGCGGCGACACAGACAGAGCAAGGGCGCGGCGGATC   (-43)  AUG
Human L-Fer    (3)     GTTCGGCGGTCCCGCGGGTCTGTCTCTTGCT---TCAACAGTGTTGGACGGAACAGATC    (-139) AUG
Human H-Fer    (5)     TCTTCGCCGAGAGTCGTCGGGGTTTCCTGCT---TCAACAGTGCTTGGACGGAACCCGGC   (-149) AUG
Consensus                             G-----G-------C---------CA--G---GG-CG---C----C
                                      *             *         **  *   ** **
```

Conserved C   +76   82-87   Loop (wrt human APP mRNA)
+33   39-44   Loop (wrt human L-Ferritin mRNA)

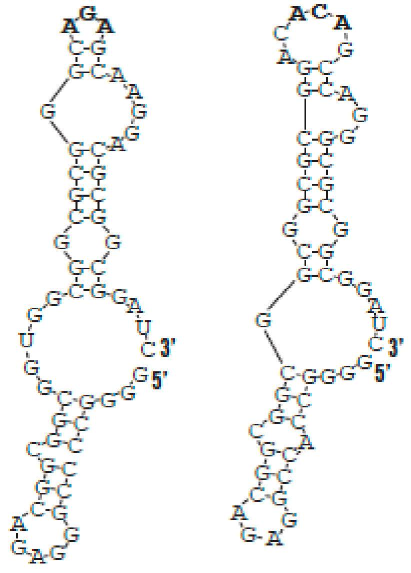

***Homo sapiens* APP**   ***Mus musculus* APP**

A. *Homo sapiens* 5' UTR Layouts

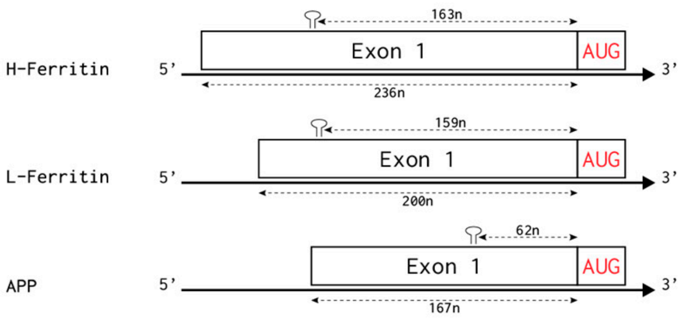

Supplemental Figure 3: Homology between the APP IRE-Type-IIs in the human, mouse, rhesus-monkey and bovine genomes relative to the canonical IREs in human L-and H-ferritin mRNAs.

RNA stem loops folded from the modified IREs folded from the human and mouse APP 5'untranslated regions.

Adapted from Rogers et al, 2016, JOURNAL OF NEUROCHEMISTRY | 2016 | 138 |479–494
